# Tertiary Endosymbiosis in Two Dinotoms Has Generated Little Change in the Mitochondrial Genomes of Their Dinoflagellate Hosts and Diatom Endosymbionts

**DOI:** 10.1371/journal.pone.0043763

**Published:** 2012-08-20

**Authors:** Behzad Imanian, Jean-François Pombert, Richard G. Dorrell, Fabien Burki, Patrick J. Keeling

**Affiliations:** Department of Botany, Canadian Institute for Advanced Research, University of British Columbia, Vancouver, British Columbia, Canada; University of Melbourne, Australia

## Abstract

**Background:**

Mitochondria or mitochondrion-derived organelles are found in all eukaryotes with the exception of secondary or tertiary plastid endosymbionts. In these highly reduced systems, the mitochondrion has been lost in all cases except the diatom endosymbionts found in a small group of dinoflagellates, called ‘dinotoms’, the only cells with two evolutionarily distinct mitochondria. To investigate the persistence of this redundancy and its consequences on the content and structure of the endosymbiont and host mitochondrial genomes, we report the sequences of these genomes from two dinotoms.

**Methodology/Principal Findings:**

The endosymbiont mitochondrial genomes of Durinskia baltica and Kryptoperidinium foliaceum exhibit nearly identical gene content with other diatoms, and highly conserved gene order (nearly identical to that of the raphid pennate diatom Fragilariopsis cylindrus). These two genomes are differentiated from other diatoms' by the fission of nad11 and by an insertion within nad2, in-frame and unspliced from the mRNA. Durinskia baltica is further distinguished from K. foliaceum by two gene fusions and its lack of introns. The host mitochondrial genome in D. baltica encodes cox1 and cob plus several fragments of LSU rRNA gene in a hugely expanded genome that includes numerous pseudogenes, and a trans-spliced cox3 gene, like in other dinoflagellates. Over 100 distinct contigs were identified through 454 sequencing, but intact full-length genes for *cox1*, *cob* and the 5′ exon of *cox3* were present as a single contig each, suggesting most of the genome is pseudogenes. The host mitochondrial genome of K. foliaceum was difficult to identify, but fragments of all the three protein-coding genes, corresponding transcripts, and transcripts of several LSU rRNA fragments were all recovered.

**Conclusions/Significance:**

Overall, the endosymbiont and host mitochondrial genomes in the two dinotoms have changed surprisingly little from those of free-living diatoms and dinoflagellates, irrespective of their long coexistence side by side in dinotoms.

## Introduction

Reduction is a universal theme in the symbiotic events that gave rise to mitochondrial and plastid diversity. In primary endosymbiosis, the α–proteobacterial and cyanobacterial ancestors of mitochondria and plastids were drastically reduced to organelles that encode only a small fraction of their original genes [1]–[4]. In plastid evolution, this was followed by further rounds of primary and secondary endosymbiosis. Secondary endosymbionts, derived from red or green algae, have also lost nearly everything except their plastids [5], [6], and even in those exceptions where secondary endosymbionts retained a miniature nucleus (nucleomorph), it is highly reduced and nearly all its cytoplasmic features are gone [7]–[11]. In tertiary endosymbionts generally only the plastids remains [12], with one interesting exception, the so-called ‘dinotoms’.

With 10 known species, dinotoms are a small group of closely related dinoflagellates whose endosymbionts are thought to belong to at least three different diatom clades [13]–[17]. Considering the small size of this group, dinotoms are very diverse in their morphologies (for example, with or without thecal plates with different plate configurations among the thecate species), their habitats (fresh water or marine environments), and their life styles (planktonic or benthic, dominantly motile or prevailingly sessile), and have consequently been classified into five distinct genera.

The tertiary diatom endosymbiont of dinotoms has, like other tertiary endosymbionts' reduced to some degree: it has lost its distinctive cell wall, motility, and the ability to divide mitotically [18], [19]. Despite these losses and integration within its host, however, the endosymbiont has also retained many of its original characters, including a large nucleus with vast amounts of DNA, a large volume of cytoplasm separated from the host by a single membrane, and perhaps most surprisingly its own mitochondria [20]–[25].

In two dinotom species, Durinskia baltica and Kryptoperidinium foliaceum, it has been shown that the mitochondria of the endosymbionts still express genes for cytochrome c oxidase subunit 1 (cox1) and cytochrome b (cob) [21], [26]. The host mitochondria in D. baltica also expresses cox1 and cob, so this species at least is thought to possess uniquely redundant mitochondria [21], [27]. While diatom and dinoflagellate mitochondria are similar morphologically, they could not be more dissimilar in terms of genomic content and organization. Sequenced diatom mitochondrial genomes range from 43 to 77 kbp, have a circular map, and encode about 60 genes. While generally compact, they usually feature one large intergenic spacer composed of repetitive sequences (from nearly 5 kbp in the centric diatom Thalassiosira pseudonana and the araphid pennate diatom Synedra acus, to about 35 kbp in the raphid pennate diatom Phaeodactylum tricornutum) [28], [29]. In contrast, dinoflagellate mitochondria encode only three protein-coding genes (cox1, cox3 and cob) and many fragments of ribosomal RNA (rRNA), and these appear to be organised on multiple chromosomes that may be linear, and which are greatly expanded in number and include numerous incomplete copies or pseudogenes along with highly dispersed short or long stretches of non-coding and repetitive sequences [30]–[32]. The disposal of the canonical start and stop codons of the 3 protein-coding genes, trans-splicing of *cox3* in at least a few species, polyadenylation and editing of the mitochondrial transcripts are among other oddities observed in the dinoflagellate mitochondrial genomes [30]–[33].

The co-occurrence of these two distinct mitochondria within dinotoms raises questions about whether or not either or both genomes have been reduced in any way due to this unique mitochondrial redundancy; or more specifically, do host and symbiont mitochondrial genomes encode a similar suite of genes found in mitochondria of free-living diatoms and dinoflagellates that lack a symbiont? In endosymbiotic partnerships, the symbiont is generally the more reduced, so it is of interest to know whether the dinotom symbiont have retained a full suite of diatom mitochondrial genes or not. However, in this case the host genome is also of interest because dinoflagellate mitochondrial genomes are already highly reduced so that all the genes they originally encoded are also found in the symbiont. To address these questions and investigate the outcome of the permanent and obligate tertiary endosymbiosis on the content and organization of the two distinct mitochondrial genomes in dinotoms, we sequenced the endosymbiont mitochondrial genomes of D. baltica and K. foliaceum. We also extensively sequenced the *D. baltica* host mitochondrial genome (but not completely since the nature of dinoflagellate mitochondrial genomes is not compatible with ‘complete’ sequencing), and produced the first sequencing data from the host mitochondrial genome in *K. foliaceum* in addition to extra sequencing data pertaining to the transcription in both genomes. Then, we compared these data from endosymbiont and host in dinotoms with available diatom and dinoflagellate mitochondrial genomes and sequences, respectively, to see if they are in any way reduced in relation to their free-living counterparts. We find both endosymbiont genomes are almost identical in gene content to other diatoms and even genome organization is almost identical to that of the raphid pennate diatom Fragilariopsis cylindrus. We also find that the host mitochondrion in D. baltica encodes complete copies of cox1and cob genes and a bipartite cox3 gene, many pseudogenes of all three genes, along with several fragments of the large subunit of ribosomal RNA gene (LSU rRNA), exactly as described in other dinoflagellates [30]–[33]. From the host mitochondrion in K. foliaceum, we also characterized the first identified fragments of the three protein-coding genes, their corresponding transcripts along with the transcripts of several LSU rRNA fragments, all of which show a high degree of homology with their counterparts in other dinoflagellates. Overall, it appears that the endosymbiotic integration of the diatom with its dinoflagellate host has had no detectable effect on the evolution of its two distinct mitochondrial genomes, which contrasts with all other secondary and tertiary endosymbionts, where the organelle is lost altogether.

## Results

### The endosymbiont mitochondrial genomes of D. baltica and K. foliaceum

From the A+T-rich fraction of DNA of D. baltica and K. foliaceum, 299 and 635 pyrosequencing reads with an average length of 366 bp and 386 bp were respectively identified as endosymbiont mitochondrial sequences. A total of 169 and 123 Sanger reads were also used in the assemblies, resulting in single contigs of 35,505 bp (D. baltica) and 39,686 bp (K. foliaceum) with an overall coverage of 5.46× and 7.73×, respectively. We were unable to bridge the final gap in both genomes, despite numerous attempts using different long-range PCR protocols under different conditions, buffer systems, and primers. This is most likely due to the presence of a large intervening sequence, as is common to other diatom mitochondrial genomes (for example the 35 kb insertion in P. tricornutum [29]), and/or to the presence of repetitive elements that may form complex secondary structures that inhibit PCR. Since all the other sequenced diatom mitochondrial genomes map as circular molecules [28], [29], it is likely that the D. baltica and K. foliaceum genomes share the same configuration.

### General features of the endosymbiont mitochondrial genomes of D. baltica and K. foliaceum

**Figure 1:**
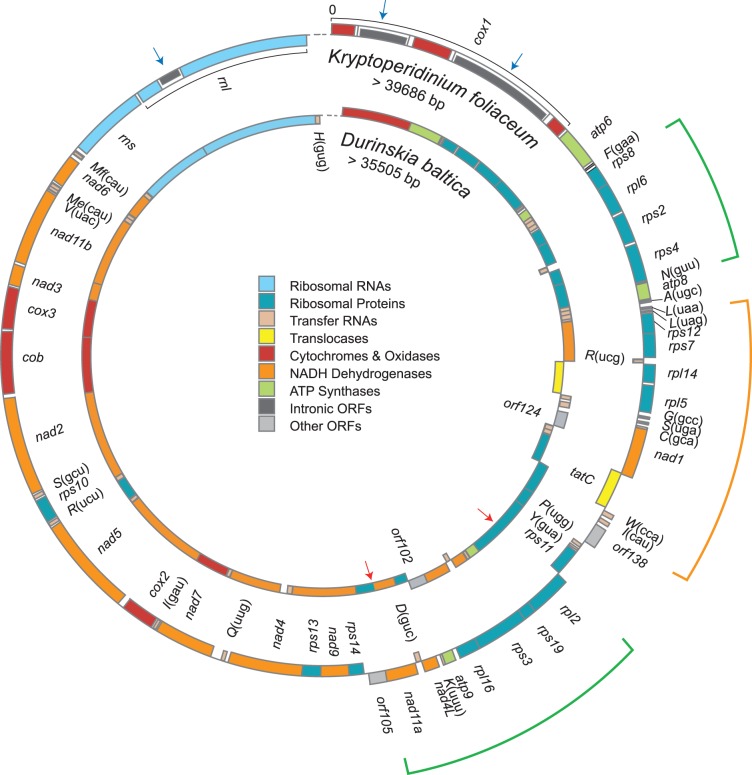
The mitochondrial genome maps of the endosymbionts in Durinskia baltica and Kryptoperidinium foliaceum. Functionally related genes are colour-coded and transcriptional direction is clockwise (boxes outside the ring) or counterclockwise (inside). Genes for tRNAs are indicated by their single letter code. The dashed lines represent the gap in the genomes. The blue arrows specify the locations of the introns in the map for K. foliaceum, and the red arrows point at the locations of gene fusions in the map of D. baltica. The arcs show the conserved gene blocks in the two dinotoms and P. tricornutum (green and orange arcs) and T. pseudonana (the green arcs). The two genomes are not represented in scale with respect to one another.

The coding regions of the endosymbiont mitochondrial genomes of D. baltica (34,242 bp) (GenBank: JN378735) and K. foliaceum (34,742 bp) (GenBank: JN378734) are very similar in size, form and content to those of other diatoms (Table 1). They are compact, featuring small intergenic spacers and a number of overlapping genes, and encode 58 and 59 genes, respectively (figure 1, Table 1). In addition to two rRNA genes, D. baltica and K. foliaceum mitochondria respectively encode 33 and 35 protein-coding, and 23 and 22 tRNA genes. Both code for the initiator and elongator methionine tRNAs but seem to lack tRNAs for threonine, like all other known diatoms and heterokonts [33]. The apparent absence of a tRNA for glutamic acid (trnE) is shared with S. acus but not with their closer relative P. tricornutum, and the histidine tRNA is missing from K. foliaceum but not D. baltica. In the latter case, it is possible that the missing tRNA genes are encoded in the unsequenced portion of the genomes, as they are encoded in other diatom mitochondria. The two dinotom mitochondrial genomes also share two potentially spurious open reading frames (ORFs) larger than 100 amino acids (aa), orf138 and orf105 in K. foliaceum and orf124 and orf102 in D. baltica, respectively displaying 67% and 55% aa identity to each other. These ORFs are not found in other diatoms and show no significant homology in BLAST searches [34]. Interestingly, the endosymbiont mitochondrial gene complement is well-conserved across the larger group of stramenopiles or heterokonts that include diatoms [35]. Gene length comparisons between the mitochondrial genes in the two endosymbionts and those of diatoms indicate that their protein-coding and rRNA genes are also very similar in size (Figure S1). Only the rpl2 gene in D. baltica seems shorter at the 5′-end, however, it still retains both the conserved RNA-binding and the C-terminal domains.

**Table 1:** General characteristics of mitochondrial genomes in dinotoms compared to diatoms.

	Durinskia baltica	Kryptoperidinium foliaceum	Phaeodactylum tricornutum	Synedra acus	Thalassiosira pseudonana
Size (bp)					
Total	>35505	>39686	77356^a^	46657 b	43827^a^
Coding and intergenic	34242	34742	35177^a^	35944 b	36519^a^
GC content (%)					
Total	31.02	32.41	35.08	31.78	30.11
rRNA genes	36.27	36.57	36.66	34.03	33.03
tRNA genes	44.03	43.72	43.01	38.52	40.55
Protein-coding genes	30.25	31.64	32.84	30.73	28.96
Intergenic spacer	22.14	26.15	26.17^c^	26.74	23.53 d
Gene content					
Total	58	59	60	61 b	61
Protein-coding genes	33	35	34	33 b	34
rRNA genes	2	2	2	2 b	2
tRNA genes	23	22	24	24 b	25
Intronic ORFs	0	3	2	2 b	1
Other ORFs	2	2	0	3 b	0
Coding sequence (%)	90.45	83.03	77.01^e^	88.87	82.88^f^
Introns	0	3	4	3 b	1
Gene overlaps (pairs)^g^	4	2	6	1	1
Fused genes (pairs)^h^	2	0	1	0	0
Intergenic spacer (bp)	58	109	841^a^	73	157^a^
Gene length^i^	793 (554)	709 (540)	770 (538)	758(531)	741 (519)

a Data from Oudot-Le Secq and Green 2011.

b Data from Ravin et. al. 2010.

c Calculated without repeat region (with repeat region it is 36.28%).

d Calculated without repeat region (with repeat region it is 30.10%).

e Calculated without repeat region (with repeat region it is 41.72%).

f Calculated without repeat region (with repeat region it is 73.48%).

g In D. baltica: rps12-rps7, nad1-tatC, rps19-rps3-rpl16 fusion, orf124-trnP. In K. foliaceum: rps12-rps7, nad1-tatC. In P. tricornutum nad4-rps13, rps2-rps4, nad1-tatC, rpl2-rps19, rps19-rpl16, rpl5-trnG. In S. acus and T. pseudonana nad1-tatC.

h In D. baltica: rps3-rpl16, rps13-nad9. In P. tricornutum: nad9-rps14.

i First number is the average length of protein-coding genes, the number in parentheses is the average length of all genes.

The overall G+C content is very similar in the two endosymbiont mitochondrial genomes, albeit slightly less so in their intergenic regions (Table 1). Their G+C content is also consistent with that of the other diatom mitochondrial genomes, with the higher total G+C content observed in that of P. tricornutum due at least in part to the presence of a large 35 kb-long insertion (nearly half of its genome) with repetitive elements having 36.7% G+C content (33.6% GC content without). Like their pennate diatom counterparts in S. acus and P. tricornutum, the endosymbiont mitochondrial genomes of D. baltica and K. foliaceum use the universal genetic code. In contrast, the centric diatom T. pseudonana [29] and possibly two other Thalassiosirales, T. nordenskioldii and Skeletonema costatum [35] use TGA for tryptophan rather than as a signal for translational termination. In addition to the canonical ATG, the two dinotoms use ATA (rps2, rpl2, nad3 in D. baltica and atp8 in K. foliaceum) and ATT (rps2 in K. foliaceum) as alternative start codons. The alternative start codons are utilized by other organisms including diatoms. S. acus, for example, uses GTG (tatC, nad5 and cox2), P. tricornutum uses TTG (cox3, cob and tatC) and GTG (nad7), and T. pseudonana uses ATT (atp8) as alternatives for ATG. The two endosymbiont mitochondrial genomes use all the codons for their proteins just like their diatom and brown algal counterparts [36], hence the missing tRNAs must be imported from cytosol. As with most A+T rich genomes, D. baltica and K. foliaceum endosymbiont mitochondrial genomes display a bias towards A or T in the third codon position of their protein-encoding genes (79% and 76%, respectively), as do their diatom counterparts (T. pseudonana 79%, S. acus 76%, and P. tricornutum 72%).

### Gene fission

One of the protein-coding genes, nad11, in the endosymbiont mitochondrial genomes of D. baltica and K. foliaceum is broken into two parts corresponding to its two functional domains: the iron-sulfur (FeS) binding (nad11a) and the molybdopterin-binding (nad11b) domains. These two new segments have acquired a new stop codon (nad11a) and a new start codon (nad11b) and now reside on opposite strands, distantly separated in the genome. In T. pseudonana and S. acus, nad11 remains intact. However, in the pennate diatom P. tricornutum it is divided into two segments at about the same position but on the same strand and only 13 bp apart, while in F. cylindrus nad11a and nad11b are configured exactly as in dinotoms [29]. It is noteworthy that the molybdopterin-binding domain of nad11 in brown algae is highly divergent, and has been relocated to the nucleus of at least one species, Ectocarpus siliculosus [29].

### An in-frame insertion

Another distinguishing feature of both endosymbiont mitochondrial genomes is the presence of a long insertion in nad2. This nearly 500 bp-long in-frame insertion (from amino acid 213 in both to aa 377 in D. baltica and aa 381 in K. foliaceum) is not found in P. tricornutum, S. acus or T. pseudonana, and falls within the NDH/q1-type oxidoreductase domain of the Nad2 protein, between two conserved α-helices (Figure S2). The insertion sequence shares no similarity to any known sequence, and is highly divergent between the two dinotoms: endosymbiont nad2 genes share 93% and 88% amino acid identity before and after the insertion site, respectively, whereas the inserts share only 40% identity. This insertion is not spliced at the mRNA level, as indicated by RT-PCR and sequencing.

### Gene fusions in D. baltica

The mitochondrial genome of the endosymbiont in D. baltica also contains two pairs of genes that have fused: rps3-rpl16 and rps13-nad9 (red arrows in figure 1). In both pairs, the first gene has lost its stop codon while the second has kept its first methionine. In K. foliaceum, P. tricornutum and T. pseudonana, the rps3 and rpl16 genes are adjacent but not fused, whereas in S. acus, rps3 is degenerated and remains in the genome as a pseudogene near the rpl16 gene [28]. The other two genes, rps13 and nad9, are adjacent and in close proximity in K. foliaceum but not in the other diatoms.

### Introns in K. foliaceum

**Figure 2:**
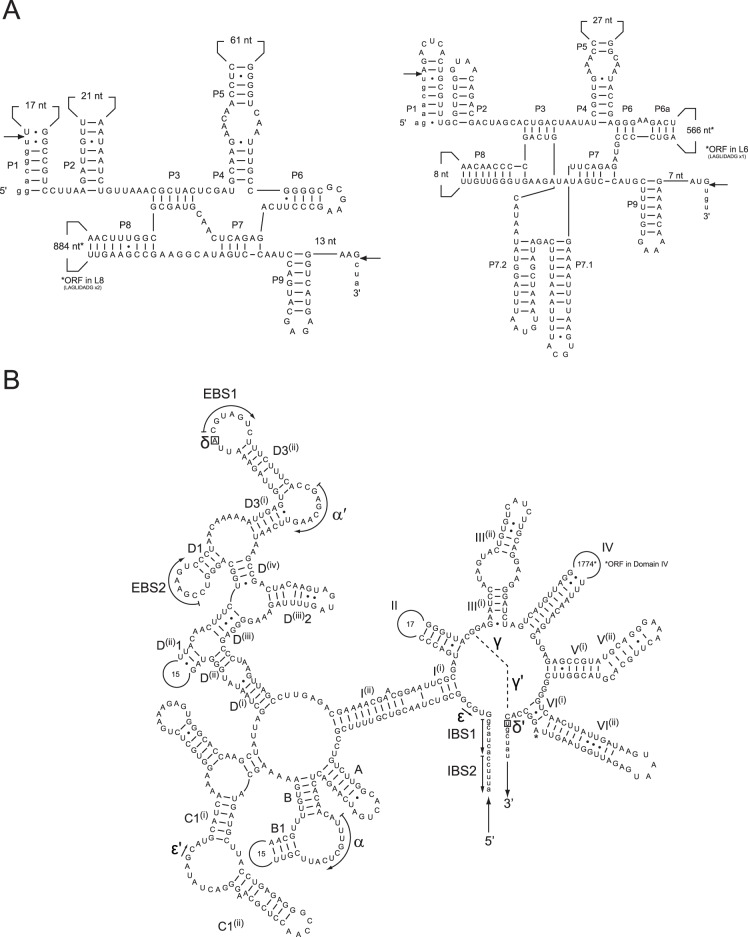
Predicted secondary structure of the three *Kryptoperidinium foliaceum* endosymbiont mitochondrial introns modeled according to the conventions described in Burke et al. [**47**] **and Michel et al.** [**48**] **.** (A) Group I introns. Left, the first cox1 intron; Right, the rnl intron. The K. foliaceum cox1 group I intron (left) had been previously mistakenly referred to as a group II intron [27]. (B) Group II intron. The second cox1 intron. Panels A and B: canonical Watson-Crick base pairings are denoted by dashes whereas guanine-uracyl pairings are marked by dots. Numbers inside variable loops indicate the sizes of these loops. Exon sequences are shown in lowercase letters. Panel A: splice sites between exon and intron residues are denoted by arrows; Panel B: the major structural domains are indicated by roman numerals and capital letters A to D, whereas tertiary interactions are represented by dashed lines, curved arrows, and/or Greek letters. Nucleotides potentially involved in the δ-δ′ interaction are boxed. Intron-binding and exon-binding sites are indicated by IBS and EBS, respectively. The putative site of lariat formation is denoted by an asterisk.

The K. foliaceum endosymbiont mitochondrion contains three ORF-encoding introns, whereas D. baltica has none. One K. foliaceum intron is found in rnl (group I) and two (group I and group II) in cox1 (figure 1 and figure 2). The orf168 located in the rnl intron codes for a putative single LAGLIDADG endonuclease while orf339 from the cox1 group I intron encodes a putative heterodimeric endonuclease carrying two LAGLIDADG motifs. The orf715 from the cox1 group II intron encodes a reverse-transcriptase maturase (RTM). Of the three K. foliaceum introns, only one is inserted at a site in common with other diatoms (Table 1): the cox1 group II intron being found in T. pseudonana and P. tricornutum, and sharing 91% and 81% nucleotide identity with the conserved cores (510 and 496 aligned residues), respectively. The K. foliaceum's orf715 is also highly similar to orf718 in the T. pseudonana intron and slightly less so with orf728, a pseudo-RTM, present in two adjacent pieces in the P. tricornutum intron (85% and 67% amino acid identity over 718 and 730 aligned residues, respectively). The close phylogenetic relationship between K. foliaceum's ORF715 and T. pseudonana's ORF718 has been corroborated independently through phylogenetic analysis [37].

### Synteny

The endosymbiont mitochondrial genomes of D. baltica and K. foliaceum are perfectly syntenic, and demonstrate striking similarity to that of the raphid pennate diatom F. cylindrus. Two large gene blocks (rps8-rpl6-rps2-rps4-trnN and rpl2-rps19-rps3-rpl16-atp9-trnK-nad4L-trnD-nad11a) are also conserved with P. tricornutum and T. pseudonana (the green arcs in figure 1), whereas a third (rps12-rps7-trnR-rpl14-rpl5-trnG-trnS-trnC-nad1-tatC-trnW-trnI) is shared with P. tricornutum (the orange arc in figure 1). With the exception of trnC, this third block is also conserved in T. pseudonana. Compared to other diatom mitochondrial genomes, there is a small inversion unique to the dinotoms (trnA-atp8).

Table 2 summarizes the estimated minimum number of inversions required for the interconversions of the diatom mitochondrial genomes. Transition from either dinotom mitochondrial genome to that of P. tricornutum, and vice versa, requires only 5 inversions while their transition to that of T. pseudonana requires 6 inversions. A minimum of 8 inversions are required to interconvert T. pseudonana with either P. tricornutum or S. acus.

**Table 2:** Number of inversions for the inter-conversions of the mitochondrial genomes of the two dinotoms and those of diatoms (predicted by GRIMM).

	D. baltica	K. foliaceum	P. tricornutum	S. acus	T. pseudonana
D. baltica	0	0	5	7	6
K. foliaceum	0	0	5	7	6
P. tricornutum	5	5	0	7	8
S. acus	7	7	7	0	8
T. pseudonana	6	6	8	8	0

### Transcription of the endosymbiont mitochondrial genes

We had previously shown that the endosymbiont cox1, cob, cox2, cox3 and rnl genes in D. baltica and K. foliaceum are transcribed with no signs of editing, that the cox1 introns in K. foliaceum are removed from its mRNA, and that cox3 and cob are transcribed as an operon in both D. baltica and K. foliaceum [21], [27]. In this study we further expanded our sampling of the transcripts of mitochondrial genes in the endosymbionts of dinotoms. Using RT-PCRs with DNase-treated total RNA and specific primers, we obtained partial nad5 and nad2 products from both genomes. We also investigated and confirmed the polycistronic transcription of the conserved gene block rps19-rps3-rpl16, which includes the rps3-rpl16 fused gene in D. baltica. All cDNA sequences were identical to their corresponding genes, consistent with the lack of editing in diatom mitochondrial transcripts as opposed to those of dinoflagellates which are heavily edited by substitutions [38].

### The mitochondrial genome of the dinoflagellate host in D. baltica

From the 454 sequencing data of the A+T-rich fraction of DNA in D. baltica, we identified more than 29,000 reads (average length of 349 bp amounting to more than 10 million bp) corresponding to putative dinoflagellate host mitochondrial sequences. These reads were subsequently assembled into hundreds of unique contigs. Of these, we further analyzed 123 high quality contigs that included 4,569 reads covering 89,634 bp of unique consensus sequences from the host's mitochondrial DNA in D. baltica, providing the most comprehensive assemblage of any dinoflagellate mitochondrial genome to date. The contigs vary in size from 210 to 2,740 bp, with an average length of 711 bp. We identified full-length copies of the cox1 and cob genes, the cox3 gene that is split into two parts (GenBank: JX001475-JX001478) along with several fragments of the large subunit ribosomal RNA (LSU rRNA) gene (GenBank: JX001584-JX001600). We have also recovered 102 contigs containing pseudogenes of cox1 (GenBank: JX001520- JX001583), cob (GenBank: JX001497- JX001519) and cox3 (GenBank: JX001482- JX001496).

### Host mitochondrial protein-coding genes, transcription and editing

The contig containing cox1 is 2,740 bp long with 99 reads (12.6× coverage), while the contig that includes cob is 2,020 bp long with 82 reads (14.2× coverage). As is the case in several other dinoflagellates [31], [39], the *D. baltica* cox3 gene is broken in two separate parts: cox3 part 1 (cox3-1) is 733 bp long with 48 reads (22.9× coverage), while the second contig, cox3 part 2 (cox3-2), is 595 bp long, with 12 reads (7.0× coverage). The 5′ end of cox1 gene is preceded by non-coding sequence with no significant homology to any known sequences. The 3′ end of the gene is followed by 81 bp, non-coding, and then, by a cob pseudogene (339 bp) and a short cox1 pseudogene (110 bp). The cob gene is also flanked by 115 bp and 259 bp non-coding sequences at its 5′ and 3′ ends, respectively, and it is followed by 2 separate cox3 pseudogenes.

In the dinoflagellate Crypthecodinium cohnii, the cox1 gene appears in multiple copies bounded by distinct flanking sequences [40]. It is also reported, though not definitively shown, that there is more than one copy of *cox1* and *cob* genes in *K. micrum* mitochondrial genome [31]. In our extensive sequencing survey and careful assembly of the host mitochondrial genome of D. baltica, we were unable to find any evidence of multiple copies of the full-length cox1 and cob genes and cox3-1, each of which appears only in one genomic context. However, the cox3-2 that encodes the short 3′ end of the gene appears in multiple contexts (see GenBank: JX001478, JX001487, JX001488, JX001494) flanked by distinct sequences like the 3′ segment of this gene in *K. micrum* [31].

The host mitochondrial protein-coding genes of D. baltica have very similar GC content to their homologs in other dinoflagellates: 33.3%, 29.8% and 28.5% GC content for cox1, cob and cox3, respectively, compared to an average of 33.2%, 29.6% and 28.4% for the same genes, respectively, in other dinoflagellates (File S1). These genes also show high degree of nucleotide and amino acid identities to their counterparts in other dinoflagellates: cox1, cob and cox3 have an average of 95%, 95% and 89% nucleotide identities and 90%, 88% and 72% amino acid identities to their homologs in other dinoflagellates (File S1).

One of the distinguishing characteristics of the mitochondrial protein-coding genes in dinoflagellates is the genes themselves do not encode canonical start and stop codons to direct the initiation and termination of translation [30], [31], [39]. The only exception to date is the *cox3* gene of the basal dinoflagellate *Hematodinium* which encodes a canonical stop codon [39], and the *cox1* gene of *C. cohnii* which seems to encode a canonical start codon [40]. In some dinoflagellates the *cox3* transcript apparently obtains a stop codon through polyadenylation, while others simply lack a stop codon [31], [39]. The cox1, cob and cox3 genes in D. baltica resemble homologs in other dinoflagellates, in lacking canonical start and stop codons as well. There is one in-frame TGA codon in the middle of cox3, but in all likelihood this is edited at the mRNA level as has been shown in the cox1 transcript of Amphidinium carterae [41], the cox3 transcript of K. micrum [31], and others [38], [42]. Indeed, TGA, which typically codes for stop and sometimes for tryptophan, is unassigned in dinoflagellates [31], [39].

The comparison between the complete cox1 gene and its nearly complete transcript (GenBank: JX001479) obtained through RT-PCR, reveals extensive substitutional editing occurring at either the first or second codon positions, resulting without exception in an amino acid change (see Table S1). Most of the edits substitute a G for an A, while some replace a T with a C or a C with a U or more infrequently a G with a C. Most of these replacements result in a conservative substitution of an amino acid (for example, an isoleucine with a valine). The number of editing sites, their codon positions and the types of edits all are consistent with those reported for other dinoflagellates [31], [38], [39], [41], [42].

A novel feature of the cob gene is the presence of a 150-nucleotide-long in-frame insert starting at amino acid 121 to 170. The insert sequences show no homology to any other sequences in the public databases except to a 69-nucleotide-long portion of another insert within a cox1 pseudogene in D. baltica (GenBank: EF434626.1). The insert is located between the two predicted transmembrane helices, conserved also in *Alexandrium catenella* and *Pfiesteria piscicida*, without disrupting them (figure S3). The RT-PCR results show that this insert is transcribed along with the flanking conserved regions of this gene and remains unedited (GenBank: JX001480) unlike other parts of the transcript that is edited in the dinoflagellate fashion [21].

The cox3 gene in the basal dinoflagellates Oxyrrhis marina and Hematodinium sp. is unbroken [30], [39], whereas in at least five other dinoflagellates it is broken into two parts, transcribed and poly adenylated separately and then trans-spliced together to produce the full-length transcript [31], [39]. In D. baltica, cox3 is similarly encoded as two separate sections. The cox3-1 segment encodes the first 705 nucleotides (corresponding to the first 235 amino acids), the 5′ end of the gene, and it is followed by 27 nucleotides of non-coding sequences. The cox3-2 encodes the 153 nucleotides corresponding to the 3′ end of the gene, and it is flanked by stretches of 297 and 145 nucleotides unrelated to cox3 sequences. In K. micrum, the trans-splicing site is predicted to occur between the codons for the amino acid 235 and 236 [31], which is the same position where the two parts are patched together in D. baltica (amino acid 235–236). The evidence for the conserved site of trans-splicing comes from the RT-PCR results. The cox3 transcript in D. baltica (GenBank: JX001481) covers the nucleotides 306 to 768 (corresponding to amino acids 102 to 258) traversing the two separate parts of the gene including their junction while there is not even a single 454 sequence (out of more than 29,000 host mitochondrial sequences we identified from the A+T-rich fraction of the DNA) that spans the two parts of the gene. The comparison between the cox3 gene and its transcript reveals extensive editing especially upstream the trans-splicing site (about 36 substitutions), which also includes five A residues at the junction site. This penta-A is also found at the junction of the two parts of the cox3 gene in K. micrum and is thought to have been derived from the poly A tail of the part one of the gene [31].

### Host mitochondrial ribosomal RNA gene fragments

The ribosomal RNA genes in both apicomplexans and dinoflagellates are highly fragmented, and 20 or more fragments have been identified in a few species from both taxa [31], [39], [43]. We have identified 8 unique fragments of the LSU rRNA in D. baltica: LSUA, LSUD, LSUE, LSUF, LSUG, RNA2, RNA7 and RNA10-like fragments. The LSUA, LSUE and RNA10-like fragments appear in two copies, each of which within a different genomic context. Compared to their homologous sequences in other dinoflagellates (for example, in K. micrum, A. catenella and P. piscicida) the D. baltica LSU rRNA fragments are highly conserved (on average between 88% to 96% nucleotide identities).

### The host mitochondrial genome is dominated by pseudogenes

**Figure 3:**
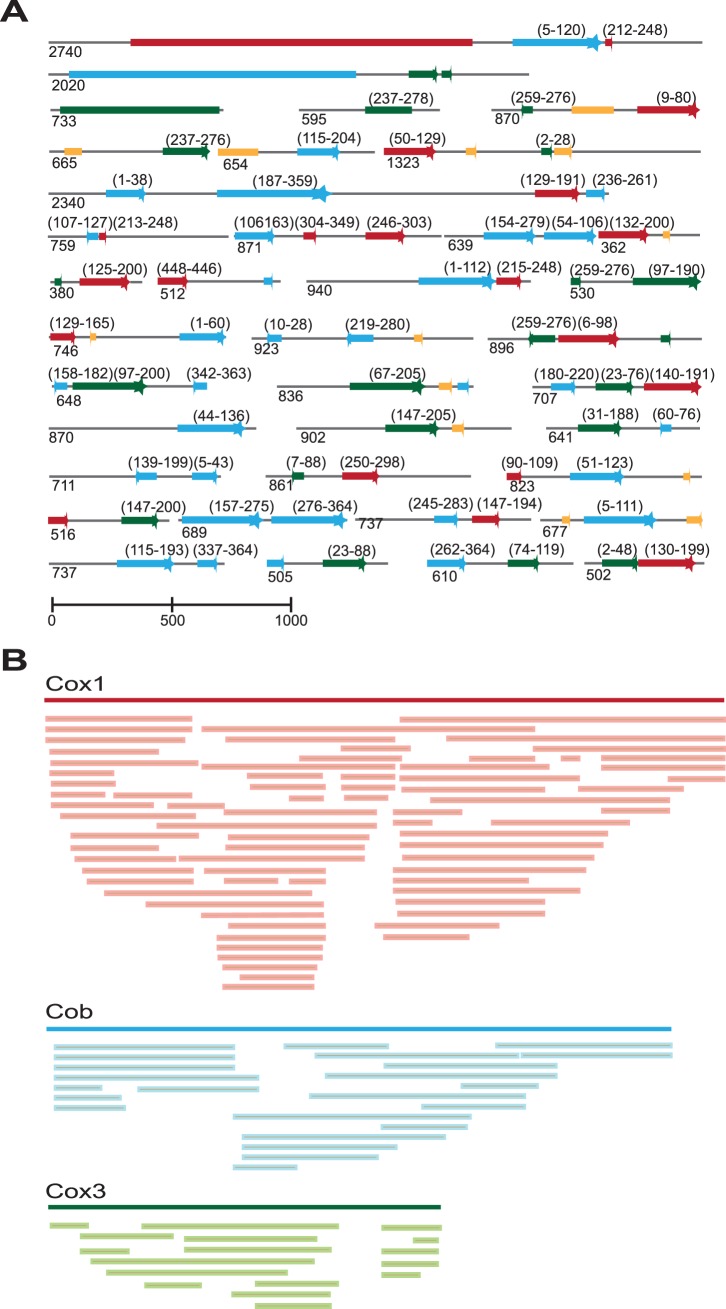
Genes and their pseudogenes in the mitochondrial genome of Durinskia baltica. (A) The full-length genes and their derived pseudogenes. The full-length protein-coding genes and the LSU rRNA gene fragments are represented by colored blocks, while the pseudogenes are shown by colored blocks with a broken tip. The lines represent non-coding sequences. The genes and their matching sequences within the pseudogenes are color-coded: cox1 in red; cob in blue; cox3 in green; LSU rRNA fragments in yellow. The sequences are drawn in scale. The numbers at the bottom of the contigs show their sizes in nucleotides, while the numbers on the top within parentheses specify the number of the first and last amino acids on the full-length gene corresponding to the conserved sequences of the pseudogenes. (B) The Alignment of the conserved regions of many pseudogenes with their corresponding full-length gene.

The mitochondrial genomes of apicomplexans are among the smallest mitochondrial genomes, encoding only 3 protein-coding genes and highly fragmented rRNA genes in a short linear chromosome (about 6 kbp). Although the dinoflagellate mitochondrial genomes seem to be as gene-poor, their genome is expanded enormously through amplification of the few genes and gene fragments they encode, generating in some species multiple copies of these genes and more often myriads of their gene fragments or pseudogenes [21], [30], [31], [39]–[41]. In this regard the mitochondrial genome of the host in D. baltica is a typical dinoflagellate mitochondrial genome with hundreds if not thousands of pseudogenes of both the protein-coding and LSU rRNA gene fragments. These pseudogenes appear in a wide variety of sizes, orientations and genomic contexts. They generally include a highly conserved portion of the true genes (usually with 99% to 100% nucleotide identity to their corresponding sequences found in the full-length genes), flanked by different non-coding and/or repetitive sequences (figure 3A). The conserved regions of these pseudogenes appear in various lengths, and we present the sequence data, for the first time, demonstrating that they are derived from all different regions of the full-length genes without any apparent preference or hot spots for any specific region (figure 3B).

Although the majority of the pseudogenes show a high degree of sequence identity to different regions of the true genes, we identified a number of pseudogenes with different degrees of degeneration. For example, a cox1 pseudogene (GenBank: JX001555) is highly conserved along the first 327 nucleotides (99% identity), but it is followed by a cob pseudogene that is highly degenerated (only 44% identity to other dinoflagellates' cob). In another example (GenBank: JX001543) a degenerated cox3 pseudogene (46% identity) is located between two conserved cob and cox1 pseudogenes. These degenerate sequences in the presence of many well-conserved gene fragments may indicate that rampant amplification and recombination not only play a role in sequence conservation of many pseudogenes [39] but also simultaneously generate many mutations elsewhere.

### The mitochondrial genome of the dinoflagellate host in K. foliaceum

While we recovered thousands of sequences with significant homology to dinoflagellate mitochondrial sequences from the A+T-rich fraction of DNA in D. baltica, we were unable to find any such sequences from the A+T-rich fraction of DNA in K. foliaceum. Our initial attempts to amplify and sequence the protein-coding genes and their transcripts using degenerate or dinoflagellate specific primers through PCR and RT-PCR, respectively, were unsuccessful. However, the 454 sequencing data from the K. foliaceum cDNA library (see Materials and Methods) generated hundreds of short sequences (average length of 76 bp) that show significant homology to mitochondrial sequences of other dinoflagellates. The assembly of these reads generated larger contigs and after subsequent PCR and RT-PCR based on these new data, we were able to recover larger fragments of all the three protein-coding genes but not their full-length sequences. These results are summarized in Table 3. We also recovered several fragments of the LSU rRNA transcripts (some in 2 copies within distinct flanking sequences) including LSUA, LSUE, LSUG and RNA7-like fragments (GenBank: JX001601-JX001608) with 358, 65, 67 and 409 pyrosequencing reads, respectively. Our attempts to recover the full-length genes and their transcripts through further PCR and RT-PCR failed. Nested primers were also tested without any results. We also tested the possibility that gene fragments were encoded on separate circular chromosomes using outward primers in PCR and long range PCR, but they did not produce any product.

**Table 3:** Partial Protein-coding genes and their transcripts found from the host mitochondrial genome of *Kryptoperidinium foliaceum*.

	GenBank Accession	Number of Contigs	Total Length (bp)	454 Reads	Sanger Reads
*cox1*	JX001614	2	968		37
*cox1* transcript	JX001613	3	1173	69	12
*cob*	JX001611	4	579		13
*cob* transcript	JX001612	3	927	105	9
*cox3*	JX001609	1	88		4
*cox3* transcript	JX001610	3	398	25	3

The host's mitochondrial protein-coding gene fragments in K. foliaceum have very similar GC content to their corresponding homologous sequences in other dinoflagellates: 34.3%, 29.6% and 28.9% GC content for cox1, cob and cox3 fragments, respectively (File S1). These gene fragments also show high degree of nucleotide and amino acid identities to their counterparts in D. baltica: cox1, cob and cox3 fragments have an average of 99%, 98% and 88% nucleotide identities and 96%, 93% and 84% amino acid identities to their homologous sequences in D. baltica (File S1).

A comparison between the cox1 gene fragments and their corresponding cDNAs reveals similar substitutional mRNA editing to those occurring in D. baltica and other dinoflagellates (see Table S1). Most of the edits affect either the first or second codon positions, resulting in an amino acid change. Just like in D. baltica, most of the edits in K. foliaceum are from A to G, but changes from T to C, C to U and G to C are also observed. Out of 11 editing sites in the cox1 mRNA of K. foliaceum 8 are conserved in D. baltica as well (Table S1).

## Discussion

### The mitochondrial genomes of the endosymbionts in D. baltica and K. foliaceum have not been reduced

The mitochondrial genomes of the tertiary endosymbionts in D. baltica and K. foliaceum share nearly all the characteristics found in mitochondrial genomes of free-living diatoms, including gene repertoire, gene length, GC content, and gene order. Their diatom gene set is also packaged in the diatom style: they are densely packed, with short intergenic sequences, a few overlapping genes, and no scattered stretches of repeated elements. The only repetitive elements in diatom mitochondrial genomes are sequestered into one or two long contiguous regions [28], [29], and it is likely that the unsequenced region of the two endosymbionts corresponds to a similar repetitive element-rich region. In short, the tertiary endosymbiosis event has had little if any effect on the endosymbiont mitochondrial genome, which is of interest since in all other comparable cases, the organelle is totally lost.

Recently, Gabrielsen et al. [44] sequenced the plastid genome of the tertiary haptophyte in the dinoflagellate Karlodinium veneficum, providing the only available haptophyte-derived plastid genome for comparison in this study. They showed that it maintains a genome, but with extensive gene losses, enlarged intergenic regions and substantial rearrangements compared to that of free-living haptophytes. Some of the existing genes in this genome have diverged so markedly that they might have become pseudogenes or reliant on RNA editing to produce functional proteins [44]. In contrast to this, we have shown that the plastid genomes of D. baltica and K. foliaceum are not reduced, and encode well-conserved genes that are organized similarly to those in the plastid genomes of free-living diatoms [45]. Moreover, the K. foliaceum plastid genome is much larger and more re-arranged, mainly because of the integration and partial maintenance of at least two relict plasmids also found in other diatoms [45].

The endosymbiont mitochondrial genomes of the two dinotoms appear equally unaffected by their integration with the dinoflagellate. Indeed, we were only able to identify a handful of features that distinguish dinotom mitochondria, or link them to a subset of free-living diatom lineages (Figure S4). First, the homologous (but divergent) long in-frame insert within nad2 is found in dinotoms but not in P. tricornutum, S. acus or T. pseudonana. Second, the dinotoms share a small unique inversion (trnA-atp8). Third, the fragmented nad11 gene and translocated nad11b is found in both dinotoms, but also in F. cylindrus [29], suggesting the dinotom endosymbionts are more closely related to this raphid pennate diatom than any other diatom for which mitochondrial genome data exist.

### The mitochondrial genomes of the host in D. baltica and K. foliaceum retain nearly all their dinoflagellate characteristics

The dinoflagellate host in D. baltica retains a typical dinoflagellate mitochondrion with tubular cristae [21], and we have shown here that this organelle maintains a genome with all the typically unusual traits of this genome in other dinoflagellates, including the gene content, the GC composition, gene and amino acid identities, abandonment of canonical start or stop codons, and genome organization [30]–[32], [39], [41]. The cox3 gene in D. baltica is encoded as two separate sections, and the transcripts are trans-spliced at the same general region of the gene in at least five other dinoflagellates (and the same nucleotide position as in *K. micrum cox3*) to produce the full-length mRNA [31], [32], [39]. Despite being gene poor the host's mitochondrial genome in D. baltica has expanded enormously through amplification and recombination, harboring numerous pseudogenes. We have also shown here that extensive substitutional mRNA editing occurs in D. baltica [31], [38], [39]. Indeed, the only novel trait we have found in the D. baltica host mitochondrial genome is the 150-nucleotide in-frame insert within its cob gene.

The mitochondrial genome of the host in K. foliaceum has been more elusive, but we have characterized several fragments of all three protein-coding genes and their transcripts along with several nearly full-length LSU rRNA fragments. These data indicate that the host in K. foliaceum has a mitochondrial genome that encodes at least the same three protein-coding genes, with very similar GC content, nucleotide and amino acid identities to those in other dinoflagellates (File S1). We have also demonstrated that the K. foliaceum cox1 mRNA editing is substitutional, and its types, codon positions, and sites show consistency with those seen in other dinoflagellates (Table S1). Overall, the data seem to be consistent with a conventional dinoflagellate mitochondrial genome in the host of K. foliaceum, though it is curiously hard to characterise.

These genomes raise the important question of why the endosymbiont mitochondrial genomes have not been completely eliminated or significantly reduced, and why the host mitochondrial genomes remain almost completely unaffected by the endosymbiosis. We have previously suggested that the mitochondrial genome redundancy (with two sets of cox1, cob and cox3 genes, one from dinoflagellate host and one from the diatom endosymbiont) found in dinotoms might be due to spatial differentiation rather than functional specialization [21]. The nearly complete endosymbiont genomes are consistent with this, but additional data from the host mitochondrial genome in K. foliaceum and from mitochondrion-targeted proteins in both nuclear genomes will be required to really determine whether the function of either organelle has been affected by the presence of the other.

### Conclusions

Despite the full integration of the diatom tertiary endosymbiont within the dinoflagellate host and the consequent unique mitochondrial genome redundancy within dinotoms, we have found no evidence of significant changes in the mitochondrial genome of the host in *D. baltica* or *K. foliaceum* compared to those in free-living dinoflagellates. Our results also indicate that the endosymbiont mitochondrial genomes in the two dinotoms closely resemble those of their counterparts in free-living diatoms, following nearly the same evolutionary path to those in other diatoms but starkly distinct from those in other secondary and tertiary endosymbionts where mitochondria are lost altogether.

## Materials and Methods

### Strains and culture conditions

Cultures of Kryptoperidinium foliaceum CCMP 1326 and Durinskia baltica (Peridinium balticum) CSIRO CS-38 were respectively obtained from the Provasoli-Guillard National Center for Culture of Marine Phytoplankton (West Boothbay Harbor, ME, USA) and from the CSIRO Microalgae Supply Service (CSIRO Marine and Atmospheric Research Laboratories, Tasmania, Australia). K. foliaceum cultures were maintained in F/2-Si medium at 22°C (12∶12 light∶dark cycle) whereas D. baltica cultures were maintained under the same conditions in GSe medium.

### Nucleic acids extraction, preparation and amplification

Exponentially growing cells were collected and ground as described previously [27]. Cells lysis, DNA extractions, precipitations, fractionations, adenine+thymine-rich (A+T-rich) DNA isolations, purifications and amplifications were performed for both species as described earlier [45]. Total genomic DNA was extracted for polymerase chain reactions (PCR) either as described previously [45], or using Master Pure Complete DNA and RNA Purification Kit (EPICENTRE Biotechnologies, Madison, WI, USA) following the manufacturer's instructions. Total RNA for RT-PCR was obtained as described earlier [27]. RNeasy MinElute Cleanup kit (Qiagen, Mississauga, ON) was used to clean up the total RNA after DNase treatment according to the manufacturer's instructions. PCR and RT-PCR reactions were performed using specific primers designed based on the obtained genomic data as described elsewhere [27], [45]. Long range PCRs were conducted either as described earlier [27], [45], or using Expand Long Template PCR System kit (Roche Applied Science, Indianapolis, IN, USA) following the manufacturer's instructions.

### cDNA construction for K. foliaceum

Approximately 5 µg of total RNA was used as template for producing cDNA with SMARTer Pico PCR cDNA Synthesis kit (Clontech, CA) according to manufacturer's protocol. In order to optimize the number of PCR cycles for our sample, we performed between 15 and 30 cycles, and, based on agarose gel, determined that the optimal amplification was reached after 18 cycles.

### Genome sequencing

The mt genomes of the endosymbionts and hosts in K. foliaceum and D. baltica and the cDNA library in K. foliaceum were sequenced using massively parallel GS-FLX DNA pyrosequencing (Roche 454 Life Sciences, Branford, CT, USA) using GS-FLX shotgun libraries prepared and sequenced at the Génome Québec Innovation Centre. Sequences were assembled de novo using gsAssembler 2.5p1 (formerly known as Newbler), edited and re-assembled with CONSED 20 [46], [47]. Gaps between contigs and ambiguous pyrosequencing homopolymer stretches were linked/ascertained by PCR and Sanger sequencing of the resulting products.

### Genome annotation and analyses

Genes were identified through BLAST homology searches [34] against the NCBI non-redundant databases [http://www.ncbi.nlm.nih/BLAST] and annotated in Artemis 12 [48]. Protein-coding genes of endosymbionts were positioned with ORFFINDER at NCBI and GETORF from EMBOSS 6.0.1 [49] and their start codons determined by orthologous comparisons with close relatives while transfer-RNA (tRNA) genes were identified with tRNAscan-SE 1.21 [50]. The 5′ and 3′ ends of the mitochondrial protein-coding genes of the dinoflagellate hosts were determined after alignments were made with those in other dinoflagellates. Ribosomal RNA (rRNA) genes of the endosymbionts were annotated after comparison with their homologs in P. tricornutum and T. pseudonana, while those of the hosts′ were annotated after comparison with their homologs in other dinoflagellates especially K. micrum, A. catenella and P. piscicida. Physical circular maps were prepared using GenomeVx [51] and refined manually. Group I and group II intron secondary structures were predicted manually according to the conventions described in Burke et al. [52] and Michel et al. [53].

Transmembrane helices domains and the insertion site in the nad2 genes and the D. baltica's cob were predicted using Domain homology searches [54], SeaView 4.0 [55] and the TMHMM Server 2.0 [http://www.cbs.dtu.dk/services/TMHMM-2.0/] [56]. Conserved gene blocks between the mitochondrial genomes of dinotoms and diatoms were identified through MAUVE 2.3.1 [57] and by manual examination of the physical maps. The hypothetical numbers of inversions between the dinotom and diatom mitochondrial genomes were estimated with GRIMM 1.04 [58].

The sequence data for F. cylindrus mitochondrial genome were downloaded through jgi website [http://genome.jgi-psf.org/Fracy1/Fracy1.download.html] and annotated as described above.

## Supporting Information

Figure S1 **Gene size comparisons between the protein-coding and rRNA genes in the two mitochondrial genomes of the dinotom endosymbionts and those of three diatoms.** Ts, *Thalassiosira pseudonana*; Sa, *Synedra acus*; Pt, *Phaeodactylum tricornutum*; Kf, *Kryptoperidinium foliaceum*; Db, *Durinskia baltica*.
(EPS)

Figure S2 **Posterior probabilities for transmembrane helices in** ***nad2*** **gene of the two endosymbionts and other diatoms.** The X-axis shows the amino acid number, and the Y-axis the probability. The two conserved transmembrane helices flanking the dinotoms' inserts are painted blue in dinotoms and diatoms.
(EPS)

Figure S3 **Posterior probabilities for transmembrane helices in** ***cob*** **gene of the host in** ***D. baltica*** **compared to that in** ***Pfiesteria piscicida*** **and** ***Alexandrium catenella*** **.** The X-axis shows the amino acid number, and the Y-axis the probability. The black arrow head marks the position of the insert within the *cob* gene in *D. baltica*.
(EPS)

Figure S4 **A few ancestral and derived characters in the mitochondrial genomes of the endosymbionts in the two dinotoms inferred based on the most parsimonious scenario.** The sequence of events is arbitrary.
(EPS)

File S1 **GC content, nucleotide and amino acid identity of mitochondrial protein-coding genes in the hosts of Durinskia baltica and Kryptoperidinium foliaceum compared to those in other dinoflagellates.**
(XLSX)

Table S1 **Editing sites on the cox1 mRNA of dinoflagellate host in Durinskia baltica and Kryptoperidinium foliaceum.**
(DOC)
