# Supplementary material for: Tertiary Endosymbiosis in Two Dinotoms Has Generated Little Change in the Mitochondrial Genomes of Their Dinoflagellate Hosts and Diatom Endosymbionts
Source: PLoS One. 2012 Aug 20;7(8):e43763. doi: 10.1371/journal.pone.0043763 (PMC3423374; doi:10.1371/journal.pone.0043763)
Supplement: Table S1 — Editing sites on the cox1 mRNA of dinoflagellate host in Durinskia baltica and Kryptoperidinium foliaceum. (DOC) [file pone.0043763.s006.doc]

Table S1: Editing sites in the *cox1* mRNA of *Durinskia baltica* and *Kryptoperidinium foliaceum*

| *Durinskia baltica* | | | | |
| --- | --- | --- | --- | --- |
| DNA Site | DNA | RNA | Codon Site | Change aa |
| **154** | **A** | **G** | **1st** | **I 🡪 V** |
| 175 | T | C | 1st | F 🡪 L |
| 305 | C | U | 2nd | S 🡪 F |
| 445 | A | G | 1st | I 🡪 V |
| 515 | A | G | 2nd | Y 🡪 C |
| 658 | A | G | 1st | I 🡪 V |
| 736 | A | G | 1st | I 🡪 V |
| 739 | T | C | 1st | F 🡪 L |
| 748 | A | G | 1st | I 🡪 V |
| 776 | T | C | 2nd | L 🡪 S |
| **998** | **A** | **G** | **2nd** | **K 🡪 R** |
| **1004** | **A** | **G** | **2nd** | **N 🡪 S** |
| **1009** | **C** | **U** | **1st** | **P 🡪 S** |
| **1012** | **T** | **C** | **1st** | **F 🡪 L** |
| **1019** | **G** | **C** | **2nd** | **G 🡪 A** |
| **1063** | **A** | **G** | **1st** | **I 🡪 V** |
| **1094** | **G** | **C** | **2nd** | **G 🡪 A** |
| 1114 | A | G | 1st | T 🡪 A |
| 1198 | G | C | 1st | V 🡪 L |
| 1211 | A | G | 2nd | N 🡪 S |
| 1225 | T | C | 1st | S 🡪 P |
| 1267 | A | G | 1st | I 🡪 V |

| *Kryptoperidinium foliaceum* | | | | |
| --- | --- | --- | --- | --- |
| DNA Site relative to *D. baltica* | DNA | RNA | Codon Site | Change aa |
| 76 | A | G | 1st | I 🡪 V |
| 90 | A | G | 3rd | I 🡪 M |
| **154** | **A** | **G** | **1st** | **I 🡪 V** |
| 676 | A | G | 1st | I 🡪 V |
| **998** | **A** | **G** | **2nd** | **K 🡪 R** |
| **1004** | **A** | **G** | **2nd** | **N 🡪 S** |
| **1009** | **C** | **U** | **1st** | **P 🡪 S** |
| **1012** | **T** | **C** | **1st** | **F 🡪 L** |
| **1019** | **G** | **C** | **2nd** | **G 🡪 A** |
| **1063** | **A** | **G** | **1st** | **I 🡪 V** |
| **1094** | **G** | **C** | **2nd** | **G 🡪 A** |

Editing sites on the *cox1* mRNA in the dinoflagellate host of *D. baltica* and *K. foliaceum* and the deduced resulting amino acid change in the Cox1 protein inferred from the differences found in the gene and its corresponding transcript sequences. The bold fonts mark the conserved changes seen in the two species.
